# Graphene as a Reversible and Spectrally Selective Fluorescence Quencher

**DOI:** 10.1038/srep33911

**Published:** 2016-09-22

**Authors:** Omer Salihoglu, Nurbek Kakenov, Osman Balci, Sinan Balci, Coskun Kocabas

**Affiliations:** 1 Bilkent University, Department of Physics, 06800, Ankara, Turkey; 2 University of Turkish Aeronautical Association, Department of Astronautical Engineering, 06790, Ankara, Turkey

## Abstract

We report reversible and spectrally selective fluorescence quenching of quantum dots (QDs) placed in close proximity to graphene. Controlling interband electronic transitions of graphene via electrostatic gating greatly modifies the fluorescence lifetime and intensity of nearby QDs via blocking of the nonradiative energy transfer between QDs and graphene. Using ionic liquid (IL) based electrolyte gating, we are able to control Fermi energy of graphene in the order of 1 eV, which yields electrically controllable fluorescence quenching of QDs in the visible spectrum. Indeed, our technique enables us to perform voltage controllable spectral selectivity among quantum dots at different emission wavelengths. We anticipate that our technique will provide tunable light-matter interaction and energy transfer that could yield hybrid QDs-graphene based optoelectronic devices with novel functionalities, and additionally, may be useful as a spectroscopic ruler, for example, in bioimaging and biomolecular sensing. We propose that graphene can be used as an electrically tunable and wavelength selective fluorescence quencher.

Colloidal quantum-dots (QDs) have attracted a tremendous amount of research interest and have a wide landscape of applications ranging from biological imaging to display technologies^1^ owing to their high quantum yields, narrow spectral emission, and photostability in ambient conditions. Besides these applications, they provide appealing optical properties to control light-matter interactions at the nanometer length scale^2^. Integration of QDs with optoelectronic devices requires an electrical means of controlling their optical properties, e.g., emission and absorption properties. Various excitation mechanisms of QDs such as optical excitations, charge injection^3^, and energy transfer^4^ have been developed for light-emitting applications^5^.

Recently, controlling intrinsic properties of QDs by electrical means has been introduced by several research groups using graphene, a single atomic layer material with excellent electrical, optical, and mechanical properties^6^. Graphene is a viable material for optoelectronics^7^ and active plasmonics^8^,^9^,^10^,^11^ owing to its electrically tunable optical properties^12^. Due to its atomic thickness and low density of states, electrostatic gating of graphene leads to large shifts in Fermi energy (E_F_). Unlike pristine graphene, which absorbs around 2.3% over a broad spectrum^13^, doped graphene has a tunable optical gap of 2E_F_ for vertical transitions due to Pauli blocking^14^. Integrating graphene on optical waveguides^15^, photonic crystals^16^ or metamaterials^17^,^18^, yields electrically tunable optoelectronic devices^19^. A new approach based on QD-graphene hybrid devices shows promises for graphene optoelectronics^6^,^20^. Placing QDs in close proximity to graphene yields unusually strong modifications in optical properties of QDs due to nonradiative energy transfer between QDs and graphene^6^,^20^,^21^. Electronic transitions in graphene mediate nonradiative energy transfer that quenches the emission of QDs depending on both the QD-graphene distance and doping level of graphene. Electrostatic doping of graphene opens up new ways to control optical properties of the coupled system. For example, Lee *et al*. demonstrated that modulating charge density on graphene using solid electrolyte, causes modulation of the emission of QDs in infrared frequencies^22^. Additionally, Tielroij *et al*. showed that tuning electrostatic doping on graphene using a back-gated transistor, yields relaxation pathways of excited erbium ions placed a few nanometer away from graphene surface^23^. On the other hand, in another recent study, static (irreversible) fluorescence quenching of QDs in the visible spectrum has been recently demonstrated by using gold nanoparticles, which are precisely placed very close to QDs by using DNA origami technology^24^. However, in previous QD-graphene studies, (i) the methods are applicable only for infrared wavelengths, (ii) device footprints are very small, (iii) spectrally selective fluorescence quenching of QDs has not been demonstrated, and (iv) controlling emission of QDs in the visible spectrum has been a challenge due the requirement of large charge densities, which yields Fermi energy around 1 eV. In this study, performing ionic liquid (IL) based electrolyte gating and designing clever device layouts in large area, we demonstrated gate-tunable fluorescence quenching of QDs in the visible spectrum. Indeed, we demonstrated electrically controllable spectral selectivity among QDs having different emission wavelengths.

## Results and Discussion

**Figure 1:**
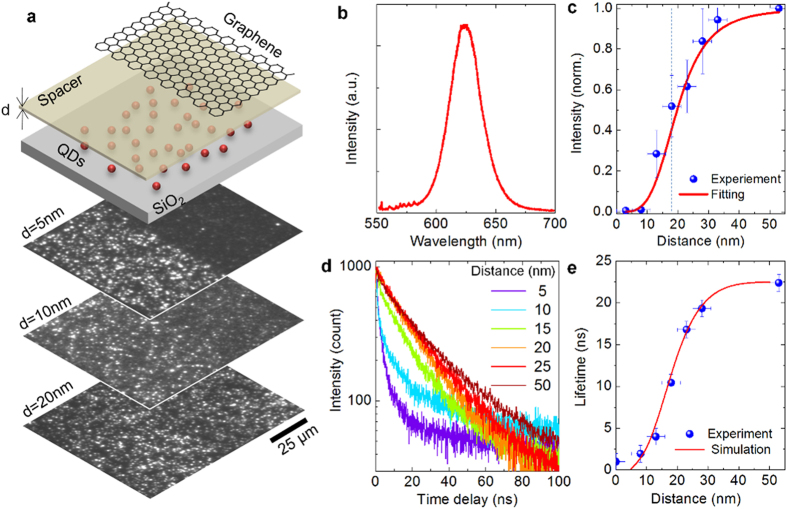
Irreversible fluorescence quenching of QDs. (**a**) The schematic shows exploded views of the samples used to measure fluorescence quenching of QDs near graphene surface. The stacked images show the fluorescence of QDs with various spacer layer thicknesses. Half of the samples are covered with graphene. (**b**) Emission spectra of QDs. (**c**) Relative fluorescence intensity of the QDs versus the QD-graphene distance. The solid line shows the fitting results using Förster-type energy transfer model. The dotted line indicates the Förster critical distance (d_0_ = 18 nm) obtained from the fitting. (**d**) Lifetime trajectories of QDs for various spacer thickness. (**e**) Dependence of the measured lifetime on QD-graphene distance. The solid line shows the fitting results.

A schematic view of the sample structure used to investigate static fluorescence quenching of QDs on pristine graphene surface is depicted in Fig. 1a. A sub-monolayer of QDs (CdSe/ZnS with emission maxima of ~625 nm) were deposited on the SiO_2_ layer by drop casting. The average diameter of QDs with the polymer cladding layer is around 6 nm (Supplementary Figure S1). Graphene was transfer-printed on the dielectric layer (Si_3_N_4,_ deposited by chemical vapor deposition, Supplementary Figure S2) which works as a spacer layer to control the QDs-graphene distance. Additionally, we patterned 50 μm wide graphene ribbons by etching graphene layer in an oxygen plasma. The florescence images (Fig. 1a) and emission spectra of QDs (Fig. 1b) were recorded for each studied spacer layer thicknesses. The stacked images in Fig. 1a show the fluorescence images of QDs for various dielectric spacer layer thicknesses. The border line between the graphene coated regions and bare dielectric regions are easily discernable from the fluorescence contrast images (Supplementary Figure S2). The mean fluorescence intensities under graphene, *I*_*G*_, and bare dielectric, *I*_*0*_, were measured by integrating the fluorescence images from 50 × 50 μm^2^ area. The scattered plot in Fig. 1c shows the dependence of relative fluorescence intensity (*I*_*G*_*/I*_*0*_) on the QD-graphene distance, which is varied from 3 nm to 50 nm. The distance-dependence of relative intensity can be modelled by Förster type energy transfer between graphene and quantum dot with a rate, *k*_*ET*_, given by:


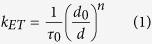


where *τ*_*0*_ is the lifetime of the QD in vacuum, *d*_*0*_ is the Förster critical distance, and *d* is the QD-graphene distance^25^. The exponent, *n*, depends on the dimensionality of the acceptor. In the case of graphene^26^, the exponent is 4. The red solid curve in Fig. 1c indicates the fit of the equation:


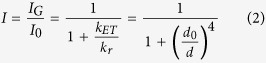


where, *k*_*r*_ is the rate of energy transfer through radiation. The fit yields the Förster critical distance of *d*_*0*_ = 18 ± 1 nm, which is relatively large in comparison to the critical distance between two dipole emitters (*d*_*0*_ = 6 nm) and comparable with the critical distance between a dipole and 2-D surface (*d*_*0*_ = 25.5 nm)^27^. Similar type of scaling behavior can be observed for the fluorescence lifetime of the QDs. Time-correlated single photon counting experiments were performed in order to measure the excited state lifetime of QDs near pristine graphene surface. As lifetime trajectories for various QD-graphene distances are shown in Fig. 1d, the fluorescence blinking of QDs results in multi-exponential behavior in the fluorescence decay. The QDs lifetime were extracted by fitting the lifetime trajectories using stretched exponential decay model^28^ as


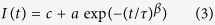


where *a* and *c* are the fitting constants, *τ* is the lifetime, and *β* is the stretched exponential parameter. The extracted lifetimes (Fig. 1e) vary from 1ns (limited by the resolution of the instrument) to 22.5 ns as the QD-graphene distance is increased from 3 nm to 50 nm. The fit gives stretched exponential parameter ranging from 0.6 to 0.8 depending on the fluorescence intensity.

**Figure 2:**
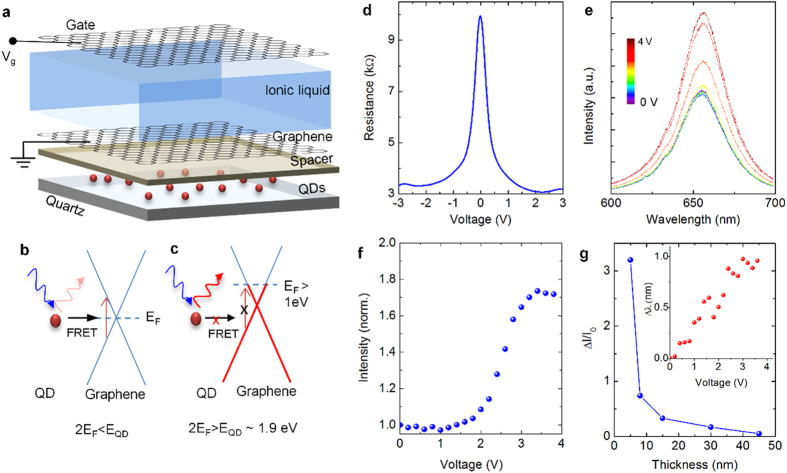
Reversible fluorescence quenching of QDs. (**a**) Schematic exploded view of the electrolyte-gated graphene transistors used for controlling fluorescence quenching of QDs via blocking of the interband electronic transitions in the visible spectrum. (**b**,**c**) Schematic representation of gate-controlled resonance energy transfer between QDs and graphene at low (2E_F_ < E_QD_) and high doping (2E_F_ > E_QD_) levels, respectively. (**d**) Variation of the resistance of graphene electrode with the gate voltage. The measured resistance also includes contact resistance of around 1 kΩ. (**e**) Recorded spectra of the emission of QDs at 655 nm for various gate voltages. (**f**) The variation of the fluorescence intensity of QDs with the gate voltage. (**g**) The variation of the change in the fluorescence intensity as a function of dielectric thickness. The inset shows the variation of the center wavelength of the QD photoemission.

Now, we would like to study gate-controlled fluorescence quenching of QDs by electrostatic doping of graphene. To elucidate this process, we developed an electrolyte gating scheme (graphene capacitor structure) which gives Fermi energies as large as 1.2 eV^14^. The main advantage of the developed device geometry is that it provides very large doping levels with optically accessible geometry. The electrolyte-gated devices consist of QDs, dielectric spacer layer (~5 nm), two graphene electrodes, and ionic liquid electrolyte as shown in Fig. 2a. Figure 2b,c illustrate the process of gate-controlled fluorescence quenching of QDs. At low Fermi energies (2E_F_ < E_Q_), the fluorescence of QDs is greatly quenched mainly due to the nonradiative energy transfer between graphene and QDs mediated by interband electronic transitions of graphene. When graphene is heavily doped (2E_F_ > E_Q_), the suppression of interband transitions results in a significant increase in the intensity of the QDs photoluminescence. During the doping process of graphene, the resistance of graphene varies from 10 to 3 kΩ as the gate voltage is scanned from 0 to +3 V, Fig. 2d. Concurrently, we measured the photoluminescence spectra of QDs under the graphene layer as a function of gate voltages. Indeed, as we increase the gate voltage, the emission intensity stays constant until a threshold voltage of about 2 V and then the intensity increases in a step-like fashion as gate voltage is increased further. The dependence of the photoluminescence intensity on the gate voltage is depicted in Fig. 2e. Consequently, we observed around 70% increase in the QD emission intensity. Although the device shows symmetric resistance variation for negative voltages, we observed asymmetric behavior in the fluorescence change. The fluorescence intensity increases more than 2-fold after a large threshold voltage of 4 V. This asymmetric behavior is likely due to the asymmetric doping of electrolyte. We observed much consistent results in the positive voltages. We repeated these experiments with thicker dielectric spacer layer and plotted the variation of fluorescence intensity as a function of spacer layer thickness as depicted in Fig. 2g. For 5 nm thick spacer layer, we observed 3-fold increase in the emission intensity; however, for 45 nm thick spacer layer, we did not observe a substantial variation in the emission intensity. It is also worth notifying here that the emission wavelength of the QDs slightly changes from 655 to 656 nm (see the inset in Fig. 2g), which is most likely due to a very small variation in the effective dielectric constant of the composite medium.

**Figure 3:**
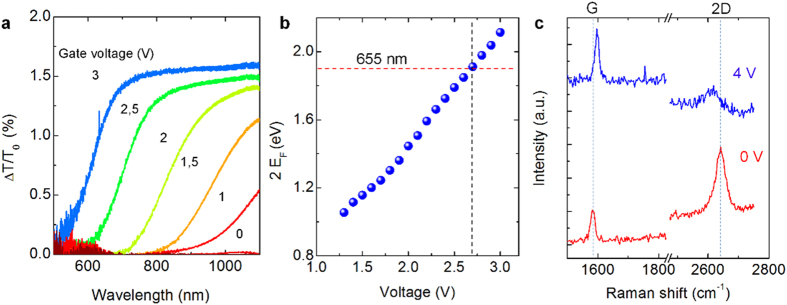
Optical characterization of highly doped graphene. (**a**) Variation of the transmittance spectra of graphene owing to the Pauli blocking of interband transitions at various gate voltages from 0 to 3 V. (**b**) The extracted Fermi energy from the step-like transmittance spectra with cut-off wavelength at 2E_F_. The red line indicates the QDs emission energy (~2 eV) and the black dash line shows the required gate voltage (~2.8 V) to block the energy transfer between QDs and graphene. (**c**) The Raman spectra of graphene at gate voltages of 0 V and 4 V. The Raman spectra were recorded using 632 nm laser. To guide the eye, the lines indicate the Raman frequency of the G ad 2D bands at 0 V.

The Fermi energy of graphene provides a useful piece of information about the mechanism of gate-controlled fluorescence quenching. We extracted the Fermi energy of the doped graphene from the optical transmittance spectra. The absorption of single layer undoped graphene on a dielectric substrate is smaller than the theoretical value of 2.3% as 
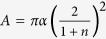
 where α is the fine structure constant, *n* is the index of refraction of the substrate^29^. On a glass substrate (*n* = 1.5), the absorption of graphene is around 1.5% of the incidence light in the visible spectrum. However, as the Fermi energy increases, the transmittance of graphene increases due to the blocking of interband transitions which results in a step-like spectrum with cut-off wavelength of 2E_F_, Fig. 3a. We observed a very clear step-like 1.6% variation of the transmittance, which agrees very well with the theoretical calculations. The cut-off wavelength directly provides the Fermi energy. Furthermore, as shown in Fig. 3b, we plotted the variation of the Fermi energies against the gate voltage. The 2E_F_ reaches the energy of QDs (2 eV) at a gate voltage of 2.8 V. In fact, this gate voltage agrees very well with the threshold voltage that we observed in the fluorescence measurements. Besides, the variations in optical transmittance, which is due to the blocking of interband transitions are altering the Raman spectra of graphene. Raman spectra of undoped and doped graphene at gate voltages of 0 and 4 V, respectively, are shown in Fig. 3c. It should be noted that, in Raman spectroscopy, we used 632 nm laser, which has similar energy with the QDs. Similarly, when we applied gate voltages larger than 3 V, we observed a rapid decline in the intensity of the 2D peak and an increase in the intensity of the G peak. As we increase the gate voltage, the frequencies of 2D and G band move to the lower and higher energies, respectively. These frequency changes are associated with the interference of possible quantum pathways of inelastic light scattering on graphene^30^. Our results suggest that electrolyte gating of graphene provides a very efficient means of controlling not only optical properties of graphene but also QDs coupled to graphene in the visible spectrum. The operation wavelength of gate tunable quenching is limited by the electrochemical window of the electrolyte. By optimizing the electrochemistry of the ionic liquid, one can reduce the operating wavelength further down to UV range.

**Figure 4:**
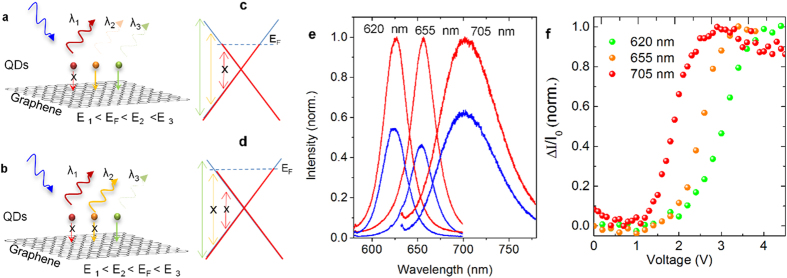
Reversible and spectrally selective fluorescence quenching of QDs. (**a**,**b**) Schematic representation of fluorescence quenching of multiple QDs on graphene surface with different Fermi energies. (**c,d**) The band structure of graphene with different Fermi energies. The arrows represents the interband transitions of different QDs. The length and color of the arrows represent their energies. (**e**) The recorded photoluminescence spectra of various QDs with emission wavelengths of 625 nm (2 eV), 655 nm (1.89 eV), and 705 nm (1.75 eV) at the gate voltages of 0 V (blue lines) and 4 V (red lines). The spectra are normalized by the intensity at 4 V for each QDs. (**f**) The variation of photoemission intensity of QDs as a function of the applied gate voltages.

In order to show the promises of our technique, we would like to generate electrically controllable spectral selectivity among quantum dots with different emission energies. Using step-like behavior of the gate-controlled fluorescence quenching in hybrid graphene-QDs devices, we can successively switch QDs at different gate voltages. We examined this process using several QDs with emission energies of 705 nm (1.75 eV), 655 nm (1.9 eV), and 625 nm (2 eV). Figure 4a–d illustrate the energy transfer mechanism between different QDs and graphene. E_1_, E_2_, and E_3_ represent energies of the QDs and E_F_ is the Fermi energy of graphene, which is reversibly tunable with the applied gate voltage. In Fig. 4e, we plotted the QDs emission spectra at gate voltages of 0 V (blue lines), and 4 V (red lines). The modulation of the fluorescence intensity of these QDs as a function of gate voltage is shown in Fig. 4f. QDs with 705 nm emission wavelength is switched on at a low gate voltage of 2.5 V where 2E_F_ matches the emission energy of the QDs. Whereas QDs with 655 nm and 625 nm emission wavelengths are switched on at gate voltages of 3 V and 3.7 V, respectively.

## Conclusion

In summary, we demonstrated reversible and spectrally selective fluorescence quenching of colloidal quantum dots placed in close proximity to graphene surface in large area. Therefore, here we, for the first time, showed that graphene can be used as an electrically tunable and wavelength selective fluorescence quencher. By tuning the Fermi energy of graphene through efficient electrolyte gating, we were able to modify the florescence intensity of QDs in the visible spectrum. Electrical control of the interband electronic transitions of graphene greatly modifies the fluorescence lifetime and intensity of nearby QDs. Ionic liquid (IL) assisted electrolyte gating enable us to control Fermi energy of graphene in the order of 1 eV yielding electrically controllable fluorescence quenching of QDs. We anticipate that gate-tunable florescence quenching together with its spectral selectivity could provide new electrical means for hybrid QDs-graphene based optoelectronic devises operating in the visible spectrum, and may be useful as a spectroscopic ruler, for example, in bioimaging and biomolecular sensing. Additionally, we anticipate that new generation of electrolytes^31^ with better charge storage capacity and large electrochemical window will directly affect the spectral selective quenching of QDs as well as fluorophores with graphene.

## Methods

### Quantum-dots

We used CdSe/ZnS core-shell quantum dots (Qdot^®^ 625, 655, and 705 Catalog Number A10197) with emission maxima of ~625 nm, 655 nm, and 705 nm, respectively, purchased from Life Technologies Corporation. The substrates were uniformly coated with QDs by drop casting the diluted ~4 nM QD solution, which was dried with a stream of nitrogen gas.

### Synthesis of Graphene

Graphene was synthesized on ultra-smooth copper foils (Mitsui mining and smelting co.,LTD, B1-SBS) by chemical vapor deposition. The partial pressure (P_CH4_ = 1.5 Torr and P_H2_ = 3.5 Torr) and flow rate of the gases (J_CH4_ = 10 s.c.c.m and J_H2_ = 85 s.c.c.m.) were used to have full coverage single layer graphene on the foils at 1035 °C. After 10 minutes of growth, the samples were left for fast cooling to room temperature.

### Patterning of Graphene

After transfer printing process, we used standard UV photolithography process for the AZ5214 photoresist and oxygen plasma etching (reactive ion etching system with 30W RF power and 20 s.c.c.m. O_2_ flow) to pattern the graphene layers.

### Dielectric Deposition

Plasma enhanced chemical vapor deposition (PECVD) was used to grow stress free silicon nitrate film on single crystal silicon (100) substrates as a gate dielectric and spacer. Growth of silicon nitrate film was carried out in PlasmaLab 8510C reactor at 200 °C and the process was carried out under the pressure of 1 Torr and RF power of 9 W. The flow rates of the gases were 25 sccm, 30 sccm, 200 sccm, and 4 sccm for N_2_, He, SiH_4_ (%2 in He), and NH_3_, respectively. The final film thickness was measured as 65 nm by an atomic force microscope and a surface reflectometer. Same process parameters with 25 °C growth temperature was used to deposit the spacer dielectric layer in order to prevent quantum dots getting damaged from high temperature process.

### Fluorescence Lifetime Measurements

Time-correlated single photon counting experiments were performed using Horiba, NanoLog series of spectra-fluorometers equipped with Fluoro3PS-Photomultiplier Power Source. NanoLED laser diode with excitation wavelength of 390 nm at repetition rates of 1 MHz was used to excite QDs. The lifetime trajectories of QDs were recorded for 400 ns time durations.

### Electrical Characterization

Electrical measurements were done by using Keithley Model 2400 and 2401 Source Meter Instrument. The measurements were performed with custom data acquisition software.

### Fluorescence Measurements

Fluorescence measurements were performed under a Nikon inverted microscope equipped with Hamamatsu EMCCD camera. To acquire simultaneous electrical and fluorescence measurements, the samples were wire-bonded on a ceramic DIP packages.

### Deposition of Spacer Dielectric Layer

Room temperature PECVD deposition was performed in order to prevent QDs to degrade from high temperature. The spacer layer deposition was carried out again in PlasmaLab 8510C reactor at 27 °C (at room temperature) and the process was done under the pressure of 1 Torr and RF power of 9 W. The flow rates of the gases were 200 sccm SiH_4_ in H_2_, and 20 sccm NH_3_. The growth rate of the dielectric was 16 nm/min.

## Additional Information

**How to cite this article**: Salihoglu, O. *et al*. Graphene as a Reversible and Spectrally Selective Fluorescence Quencher. *Sci. Rep.* **6**, 33911; doi: 10.1038/srep33911 (2016).

## Supplementary Material

Supplementary Information
