# Supplementary material for: Graphene as a Reversible and Spectrally Selective Fluorescence Quencher
Source: Sci Rep. 2016 Sep 22;6:33911. doi: 10.1038/srep33911 (PMC5031993; doi:10.1038/srep33911)
Supplement: Supplementary Information [file srep33911-s1.pdf]

Supplementary Information:

Graphene as a Reversible and Spectrally Selective Fluorescence Quencher

Omer Salihoglu[1], Nurbek Kakenov[1], Osman Balci[1], Sinan Balci[2] & Coskun Kocabas[1†]

[1]Bilkent University, Department of Physics, 06800, Ankara, Turkey

[2]University of Turkish Aeronautical Association, Department of Astronautical Engineering, 06790, Ankara, Turkey

† Corresponding author

Email: ckocabas@fen.bilkent.edu.tr

Phone: +90 312 2908078

Atomic force microscope images of colloidal quantum dots:

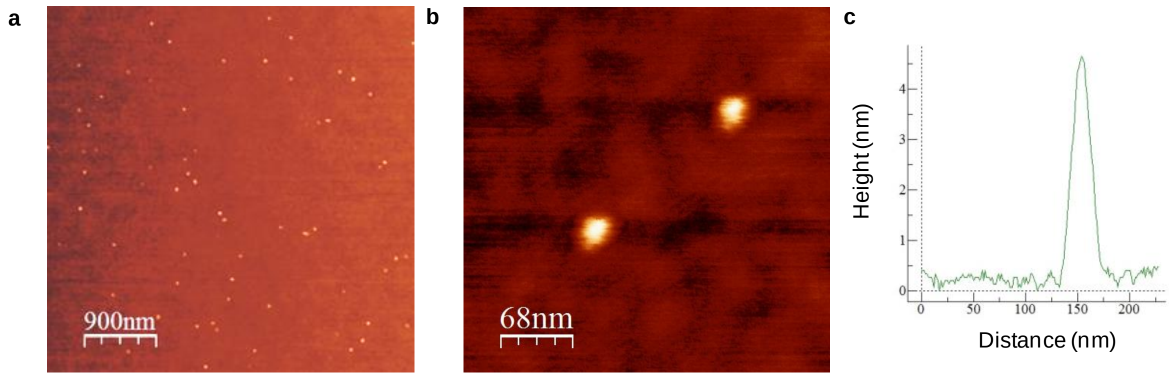

Figure S1 a-b, Atomic force microscopy images and **c**, cross-section profiles of QDs coated on Si_3N_4 dielectric using drop casting technique. The average diameter of QDs is around 6 nm. The QD-graphene distance includes the thickness of the dielectric and the radius of the QD.

Transfer-printing process of graphene:

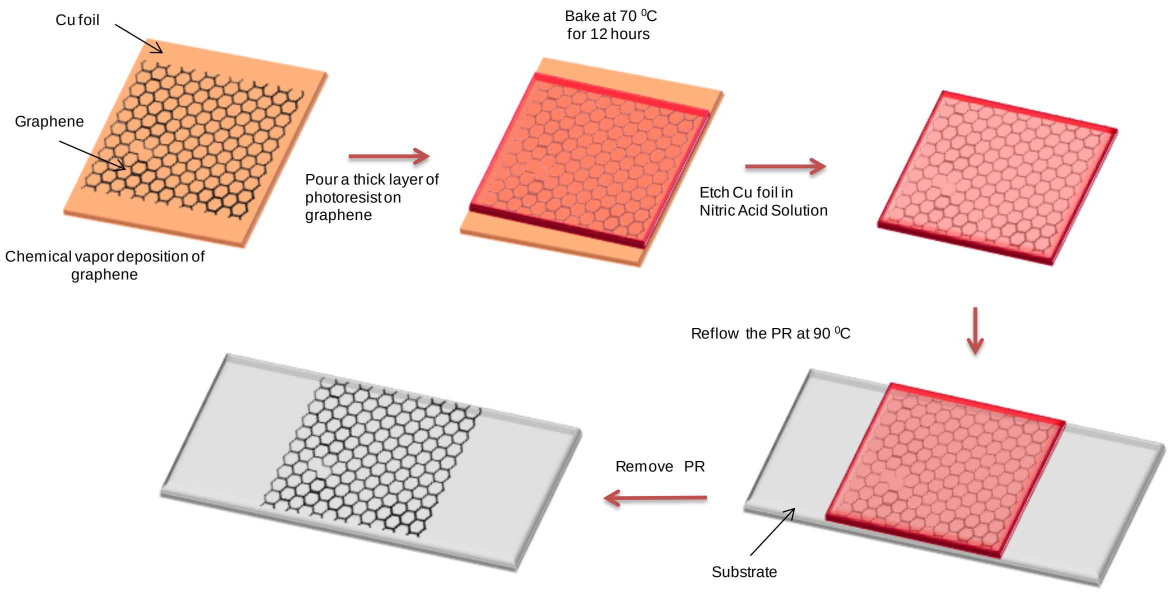

Figure S2: The steps of transfer-printing process of graphene.

After the synthesis of graphene on ultra-smooth copper foils, we used the transfer-printing process which includes the following steps:

1. Drop coat a thick photoresist layer (AZ5214) on the graphene coated surface of the copper.
2. Dry the sample in a preheated oven at 70 ^{0}C for 12 hours.
3. Insert photoresist coated sample facing copper side down on a nitric acid solution to etch copper substrate completely.
4. Rinse the etched sample with DI water.
5. Dry the samples with N_2 gas flow.
6. Locate photoresist-graphene layer on the targeted dielectric substrate with graphene side facing down.
7. Bake for 2 minutes on hot plate at 80 ^{o}C.
8. Bake for 2 minutes on hot plate at 120 ^{o}C.
9. Soak sample in acetone to remove photoresist layer.
10. Clean sample with acetone, isopropanol, and DI water and then dry with N_2 flow.

Fluoresce quenching of QDs near patterned graphene surface:

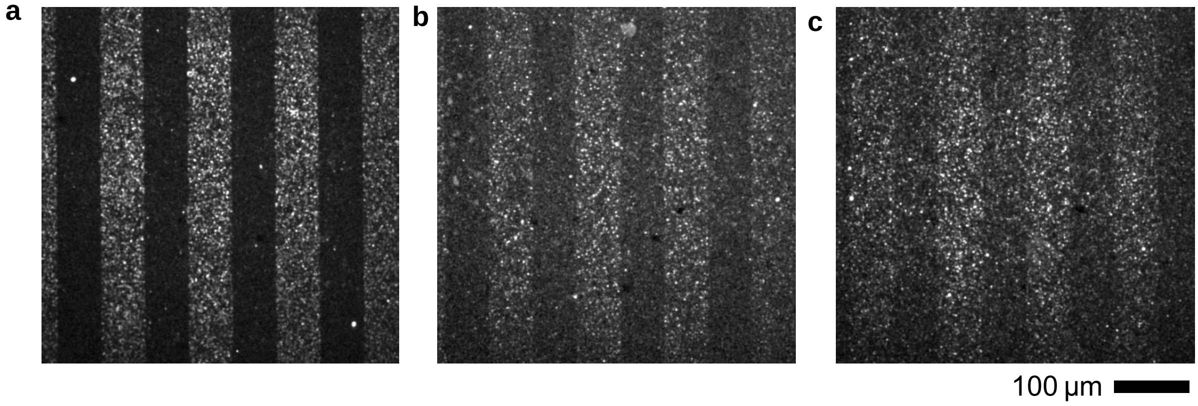

Figure S3: a-c Fluorescence microscopy images of QDs on patterned graphene for QD-graphene distance of 5, 10 and 15 nm, respectively. The fluorescence quenching is obtained from the ratio of averaged fluorescence intensities of bare and graphene coated areas.
